# Modulatory Effects of Levodopa on Cerebellar Connectivity in Parkinson’s Disease

**DOI:** 10.1007/s12311-018-0981-y

**Published:** 2018-10-08

**Authors:** Karsten Mueller, Robert Jech, Tommaso Ballarini, Štefan Holiga, Filip Růžička, Fabian A. Piecha, Harald E. Möller, Josef Vymazal, Evžen Růžička, Matthias L. Schroeter

**Affiliations:** 1 Max Planck Institute for Human Cognitive and Brain Sciences, Leipzig, Germany; 2 Department of Neurology - Center for interventional therapy of movement disorders, 1st Faculty of Medicine, Charles University and General University Hospital in Prague, Prague, Czech Republic; 3 Department of Radiology, Na Homolce Hospital, Prague, Czech Republic; 4 Clinic for Cognitive Neurology, University Hospital Leipzig, Leipzig, Germany

**Keywords:** Dopaminergic treatment, L-dopa, Levodopa, Parkinson’s disease, Resting-state magnetic resonance imaging, Eigenvector centrality, Brain connectivity, Functional connectivity, Nexopathy, Cerebellum, Cerebellar networks, Brainstem

## Abstract

Levodopa has been the mainstay of symptomatic therapy for Parkinson’s disease (PD) for the last five decades. However, it is associated with the development of motor fluctuations and dyskinesia, in particular after several years of treatment. The aim of this study was to shed light on the acute brain functional reorganization in response to a single levodopa dose. Functional magnetic resonance imaging (fMRI) was performed after an overnight withdrawal of dopaminergic treatment and 1 h after a single dose of 250 mg levodopa in a group of 24 PD patients. Eigenvector centrality was calculated in both treatment states using resting-state fMRI. This offers a new data-driven and parameter-free approach, similar to Google’s PageRank algorithm, revealing brain connectivity alterations due to the effect of levodopa treatment. In all PD patients, levodopa treatment led to an improvement of clinical symptoms as measured with the Unified Parkinson’s Disease Rating Scale motor score (UPDRS-III). This therapeutic effect was accompanied with a major connectivity increase between cerebellar brain regions and subcortical areas of the motor system such as the thalamus, putamen, globus pallidus, and brainstem. The degree of interconnectedness of cerebellar regions correlated with the improvement of clinical symptoms due to the administration of levodopa. We observed significant functional cerebellar connectivity reorganization immediately after a single levodopa dose in PD patients. Enhanced general connectivity (eigenvector centrality) was associated with better motor performance as assessed by UPDRS-III score. This underlines the importance of considering cerebellar networks as therapeutic targets in PD.

**Electronic supplementary material:**

The online version of this article (10.1007/s12311-018-0981-y) contains supplementary material, which is available to authorized users.

## Introduction

The impact of Parkinson’s disease (PD) on quality of life is severe for patients and their families [1] and goes along with a substantial economic burden that already amounts to USD 23 billion annually for the USA alone [2]. Along with the current demographic development, it is to be expected that the number of affected individuals will further increase [3]. Since its early introduction 50 years ago [4], levodopa (l-3,4-dihydroxyphenylalanine) has become the most effective and widespread symptomatic PD treatment. Though the remarkable clinical efficacy of levodopa is unquestionable, its detailed mechanisms of action in the brain are still matter of debate and its use still presents some controversies [5]. Additionally, after a prolonged treatment―usually 5 to 10 years [6]―the clinical response to levodopa progressively degrades and leads to the emergence of motor complications, such as dyskinesia and the wearing-off phenomenon [7]. Interestingly, levodopa produces both short- and long-term effects in the brain [8]. The former is associated with the immediate and dramatic motor improvement, typically observed after a single levodopa dose and is tightly linked to the drug pharmacodynamics. The latter, instead, builds up over time and is the outcome of a prolonged levodopa administration that leads to neuroplastic brain reorganization. It remains controversial whether the decline of treatment efficacy and the onset of severe dyskinesia over time are results of the progressing underlying disease or a response to direct neurotoxic effects of levodopa itself [9]. While other pharmacological as well as interventional therapies are available, levodopa remains irreplaceable in the therapeutic regimen for the majority of PD patients at least at some stages over the course of the disease. Thus, the investigation of levodopa-induced neural effects and functional brain changes using neuroimaging techniques promises to incite much needed new perspectives to optimize future therapeutic approaches for PD.

The pathophysiology of PD is characterized by a depletion of dopaminergic neurons in the substantia nigra pars compacta that leads to dopamine deficiency and promotes impairment in basal ganglia projections, in particular in the striatum [10]. From the molecular point of view, levodopa acts as a dopamine precursor protein, thus increasing dopamine availability and restoring, at least temporally, the dopaminergic signaling in the striatum of PD patients [11]. Indeed, the dopaminergic denervation of the striatum is one of the main pathological changes observed in PD. Both PD animal models and, more recently, human in vivo positron emission tomography (PET) studies with the D2 receptor agonist [^11^C]raclopride have shown the efficacy of levodopa in restoring striatal dopaminergic levels [12, 13]. However, there is no evidence that the striatum is the exclusive mediator of the levodopa response [14]; thus, a more systemic investigation of levodopa-induced brain changes is advisable.

During the last decade, resting-state functional magnetic resonance imaging (fMRI) has been implemented to investigate brain connectivity changes in PD patients [15, 16] and their modulation following levodopa administration [17]. This approach is of upmost interest, considering that many neurodegenerative disorders, including PD, have been recently conceptualized as brain disconnection syndromes or “nexopathies” [18]. However, heterogeneous results have been reported so far, likely due to the clinical heterogeneity of the studied PD cohorts and due to the methods applied for the study of brain connectivity [19].

In the present study, we investigated both general and selective functional connectivity within the motor network by means of eigenvector centrality [20] and seed-based methods. Investigating the functional brain response to levodopa may pave the way to a better understating of its therapeutic properties and mechanisms of action, possibly leading to improved treatment protocols. The aim of the present study is to shed light on the motor network functional connectivity changes following the acute administration of a single dose of levodopa in PD patients, combining resting-state fMRI and advanced data-driven graph-theory data analysis. In addition, we hypothesize an association between levodopa-induced functional connectivity modulation and clinical efficacy of levodopa.

## Methods

### Data Acquisition

In order to study the effect of dopaminergic treatment on functional brain connectivity in PD, resting-state fMRI was performed in a group of 24 PD patients (Hoehn-Yahr stages II–III, 19 males, age 55.5 ± 7.9 years (mean ± standard deviation), disease duration 12.4 ± 2.6 years, levodopa treatment duration 8.9 ± 3.1 years) in a longitudinal study design. A detailed description of the patient cohort is shown in Table 1. The selection of young patients was based on the rationale that younger PD patients show a better response to levodopa treatment [21]. Specifically, it has been reported that age is a significant modulator of the magnitude of treatment response in PD, i.e., the younger the patients, the stronger the response [22]. All procedures conformed to the Declaration of Helsinki. The study protocol was approved by the Ethics Committee of the General University Hospital in Prague, Czech Republic. All patients gave informed written consent.

**Table 1:** List of patients and demographical details

ID	Sex	Age	PD *dur*	PD *treat*	U *OFF*	U *ON*
01	M	63	15	13	21	5
02	M	53	11	7	44	9
06	M	63	14	12	21	8
07	M	53	9	8	42	11
11	M	53	12	10	37	11
13	M	45	14	6	47	21
14	M	64	13	8	31	10
15	M	53	12	9	43	10
21	M	69	9	8	47	19
23	M	49	13	12	65	18
30	M	59	9	5	38	11
31	F	44	12	3	23	4
32	M	63	11	11	33	13
33	M	60	13	9	29	17
34	M	64	17	13	36	15
35	F	70	12	5	30	13
36	F	48	13	8	28	6
37	M	55	16	10	43	23
41	M	55	12	9	46	21
43	M	60	14	14	18	8
44	F	42	9	6	33	7
45	M	55	19	15	35	5
46	M	43	9	7	34	15
47	F	50	10	6	19	6

*U Unified Parkinson’s Disease Rating Scale (UPDRS)-III score; the notation *ON* and *OFF* denotes the state of medication; age, PD disease duration (PD *dur*), and PD treatment duration (PD *treat*) are shown in years

For each patient, clinical assessment and MRI were performed in two different sessions, without dopaminergic medication and after acute levodopa challenge: *OFF* and *ON*. Four days before all measurements, dopamine agonists were substituted by equivalent doses of levodopa in each patient [23]. Other anti-PD medications (selegiline, amantadine, anticholinergics) were suspended. After an overnight withdrawal of levodopa (at least 12 h), clinical and MRI data were obtained in the *OFF* session. Clinical and imaging assessment with medication was performed in the *ON* session approximately 1 h after administration of 250/50 mg of levodopa/carbidopa, i.e., after the patient’s clinical improvement. PD symptoms were assessed with the UPDRS motor score (part III) in both sessions *OFF* and *ON*.

Functional MRI data were obtained using a 1.5-T MAGNETOM Symphony scanner (Siemens Healthcare, Erlangen, Germany) and *T*_2_*-weighted gradient echo echo-planar imaging (EPI) (repetition time, TR = 3 s; echo time, TE = 51 ms). For every patient, two fMRI scans were obtained without and with antiparkinsonian medication (*OFF* and *ON*). Each data set was acquired with 200 functional volumes and 31 axial slices (thickness = 3 mm, gap = 1 mm) with a nominal in-plane resolution of 3 × 3 mm^2^ covering the whole brain. Patients were asked to keep still, awake, and look at a fixation cross on a projection screen. For registration purposes, *T*_1_-weighted images were obtained using a magnetization-prepared rapid gradient echo (MP-RAGE) sequence (TR = 2140 ms; inversion time, TI = 1100 ms, TE = 3.93 ms, flip angle *=* 15°).

### Data Analysis

All resting-state fMRI data sets were processed using Statistical Parametric Mapping (SPM, Wellcome Trust Centre for Neuroimaging, UCL, London, UK) and Matlab® (The MathWorks Inc., Natick, MA). Standard pre-processing included realignment, slice-time correction, normalization to the Montreal Neurological Institute (MNI) space based on the unified segmentation approach [24], and spatial filtering using a Gaussian kernel with 8-mm full width at half maximum.

General connectivity was computed with eigenvector centrality (EC) using the Lipsia software package [25]. Prior to compute the EC, a baseline correction was performed using a high-pass filter with a cutoff frequency of 1/80 Hz. Thereafter, a similarity matrix was computed including Pearson’s correlation coefficient between all resting-state fMRI time courses. In particular, we used the positive correlations for EC computation in order to use a similarity matrix with only positive numbers [20]. According to the theorem of Peron and Frobenius, a similarity matrix with positive entries has a unique real largest eigenvalue, and the corresponding eigenvector has strictly positive components [26, 27]. Finally, the EC map was generated using the *i*th component of this eigenvector to obtain the EC value for voxel *i*.

After computing EC maps for all patients and both experimental sessions *OFF* and *ON*, a group analysis was performed using the general linear model with a paired *t*-test design including all 48 EC maps. Intra-individual EC differences were investigated using two different contrasts in order to check for EC increase and EC decrease with dopaminergic treatment. Resulting statistical parametric maps were processed using a voxel threshold of *p* < 0.005. In order to correct for multiple comparisons, significant clusters were obtained using the family-wise error (FWE) approach with a cluster threshold of *p* < 0.05. In addition to the parametric analysis and in order to prevent false-positive findings (see Fig. 1 in [28]), the same analysis was performed using a nonparametric approach using the threshold-free cluster enhancement (TFCE) technique [29] and the TFCE toolbox (Structural Brain Mapping Group, University of Jena, Department of Psychiatry, Germany) for SPM. This approach does not require an initial threshold to form clusters. Thus, in contrast to a two-threshold approach, TFCE is sensitive to both focal and peripheral effects. Further, as the TFCE approach does not rely on the Gaussian random field theory, it can handle images with varying local smoothness (also referred to as non-stationarity), which makes TFCE the method of choice for investigating EC differences. We used the TFCE technique with 10,000 permutations and a significance level of *p* < 0.05 (FWE-corrected).

Due to the fact that tremor plays a dominant role in cerebellar brain connectivity in PD patients [30], we also investigated a potential effect of tremor variability within our group of patients using the UPDRS-III tremor score in the *OFF* and in the *ON* state of levodopa treatment. Here, we examined the EC increase using a one-sample *t*-test across *ON*-*OFF* EC difference images. To rule out a potential influence of the tremor variability across patients, the analysis was performed using the UPDRS-III tremor score as an additional covariate of no interest. For each patient, the UPDRS-III tremor score was used in the *OFF* and *ON* state of levodopa treatment in two different analyses.

In order to detect brain regions that contribute to the intra-individual EC differences investigated above, selective connectivity was studied using seed-based correlation analyses in addition to EC mapping. Here, correlation maps were generated computing the correlation between the blood oxygenation level-dependent (BOLD) time courses of a seed voxel and all other voxels. Note that the seed voxel and also its neighbors contribute to the reference BOLD time course due to the spatial filtering during image pre-processing. All seed voxels were defined using the local maxima of the *T* scores obtained by the EC analysis described above. Correlation maps were generated for all subjects for both the *OFF* and the *ON* session and fed into a general linear model implementing a paired *t*-test. Note that such an analysis was performed for each seed voxel. Significant clusters were obtained using the same approach of cluster detection and multiple comparison correction as described above. In addition, all seed-based analyses were repeated using nonparametric tests using the TFCE toolbox as described above.

According to our main hypothesis and in order to reduce the number of statistical tests, we restricted all analysis to the motor system. Therefore, the analysis was performed in brain regions masked in a search space comprising the motor system specifically (expanded primary motor, premotor, sensorimotor cortex, basal ganglia, thalamus, brainstem, and cerebellum) based on the WFUPickAtlas. Here, we used exactly the same mask as used in a preceding letter [31]. In addition to the analysis within the mask, we also performed all seed-based connectivity analyses without using any hypothesis-driven mask in order to check the significance of our results at the whole-brain level.

### Motion Effects

Generally, head motion during MR scanning might bias the connectivity analysis and, finally, the EC values due to motion-induced signal fluctuations. This could be a particular problem if the degree of motion-related artifacts would vary between the individual scanning sessions, for example, as a consequence of treatment. Therefore, we checked for differences in head motion between both scanning sessions by computing the framewise displacement (FD) as introduced in [32]. As an input, we used the translational and rotational motion parameters obtained by SPM’s motion correction. For the whole series of 200 functional images, motion between volumes was characterized using 199 FD values for each session and subject. Finally, for each session and subject, all FD time courses were characterized by the mean FD, the maximum FD, the maximum FD after eliminating the largest 5% of the FD values, and the number of FD values exceeding 2 mm.

## Results

### Clinical Assessment

After the overnight withdrawal of dopaminergic treatment, patients showed moderate PD symptoms in the *OFF* session with a UPDRS-III score of 35.1 ± 10.8. One hour after the single dose of 250/50 mg of levodopa/carbidopa, all patients improved in the *ON* session showing less PD symptoms resulting in a decreased UPDRS-III score for all participants (11.9 ± 5.5). A paired *t*-test showed a significant decrease with *p* < 10^−10^. A more detailed analysis of PD symptoms was performed using the UPDRS-III sub-scores showing akinesia as the most prominent symptom (*OFF* 18.4 ± 5.9; *ON* 6.4 ± 3.2; *p* < 10^−10^). The other UPDRS-III sub-scores decreased also significantly: rigidity (*OFF* 8.2 ± 3.3; *ON* 2.1 ± 2.1, *p* < 10^−10^), tremor (*OFF* 2.2 ± 2.1; *ON* 0.8 ± 0.9, *p* < 0.005), and postural stability (axial; *OFF* 6.3 ± 2.7; *ON* 2.4 ± 1.1, *p* < 10^−7^).

### Brain Connectivity Analysis

**Fig. 1:**
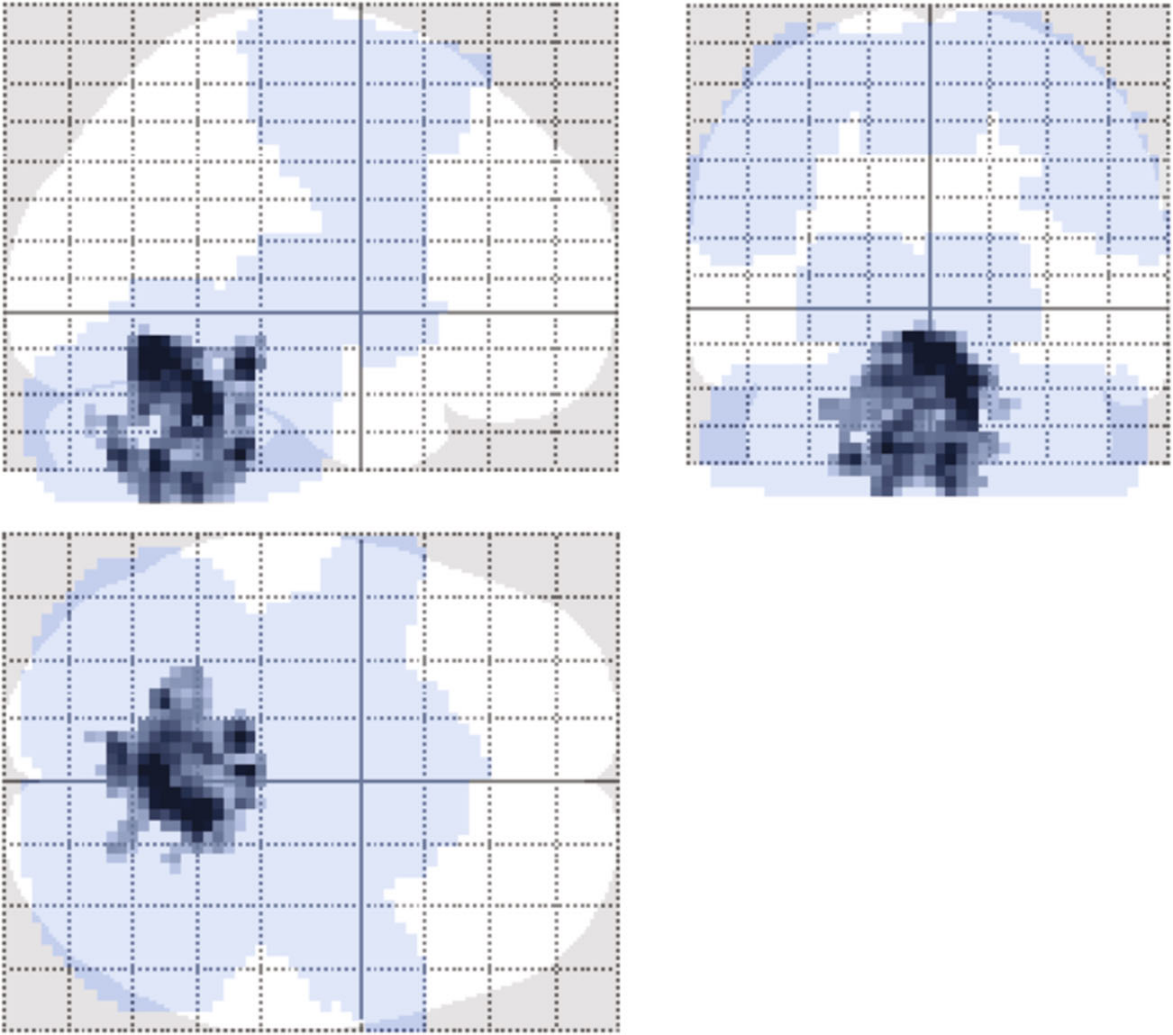
General connectivity increase in cerebellum and brainstem after levodopa in PD patients (*N* = 24) compared to *OFF* condition. Result based on eigenvector centrality analysis of resting-state fMRI restricted by a mask involving expanded primary motor, premotor, sensorimotor cortex, basal ganglia, thalamus, brainstem, and cerebellum (blue region). Gray cluster shows the pairwise *ON* vs. *OFF* difference (*p* < 0.05 with family-wise error correction at the cluster level)

Investigating the impact of dopaminergic treatment to general connectivity in PD patients, we observed a significant EC increase in cerebellum and brainstem with a pairwise comparison of the EC maps in the *OFF* and the *ON* condition. Figure 1 shows the significant result (*p* < 0.05 with FWE correction at the cluster level) using the maximum intensity projection (glass brain view). Note that we did not find any significant treatment-related EC decrease.

**Fig. 2:**
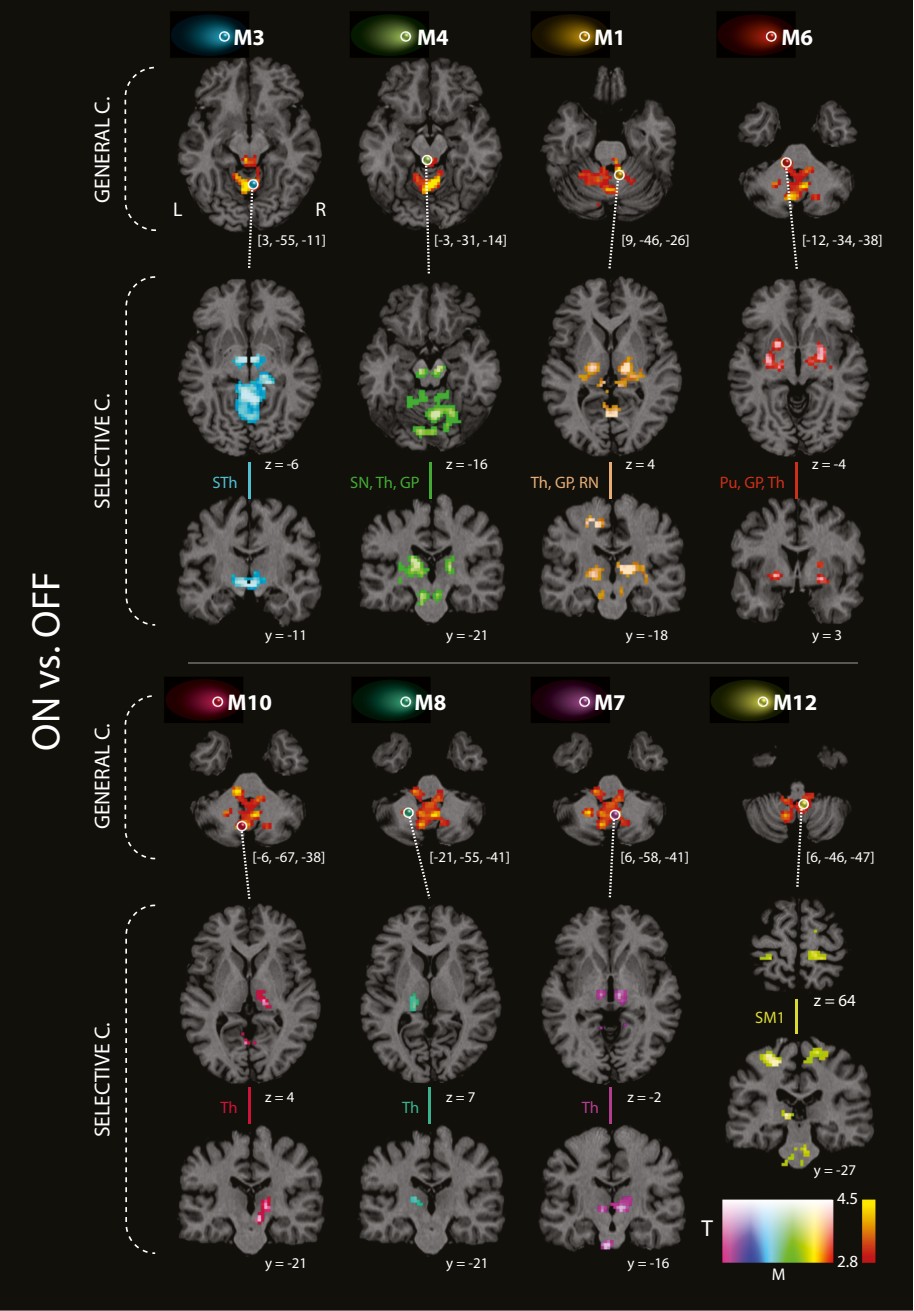
Resting-state fMRI connectivity increase of general and selective connectivity in PD patients (*N* = 24) in the *ON* compared to the *OFF* condition. The general connectivity increase (eigenvector centrality analysis; contrast *ON* vs. *OFF* condition) in cerebellum and brainstem are shown in the first and the fourth row of images (red-yellow clusters). The selective connectivity increase (correlation analysis; contrast *ON* vs. *OFF* condition) between eight seed voxels and extra-cerebellar brain structures are shown in rows 2 and 3 and in rows 5 and 6 (rainbow color clusters). The columns are sorted with respect to the *z* coordinate of each seed voxel displayed as color spheres on the general connectivity maps. Seed voxel locations and positions of the coronal and axial slices are shown using the coordinates in the MNI space. Color-coded clusters show areas with connectivity increase in the *ON* as compared to the *OFF* condition. All analyses were restricted by mask shown on Fig. 1; however, results of all seed-based correlations were also significant in full-brain analyses including family-wise error (FWE) correction at the whole-brain level (*p* < 0.05 at the cluster level). C connectivity, GP globus pallidus, Pu putamen, SM1 primary sensorimotor cortex, SN substantia nigra, STh subthalamus, RN red nucleus, Th thalamus

**Fig. 3:**
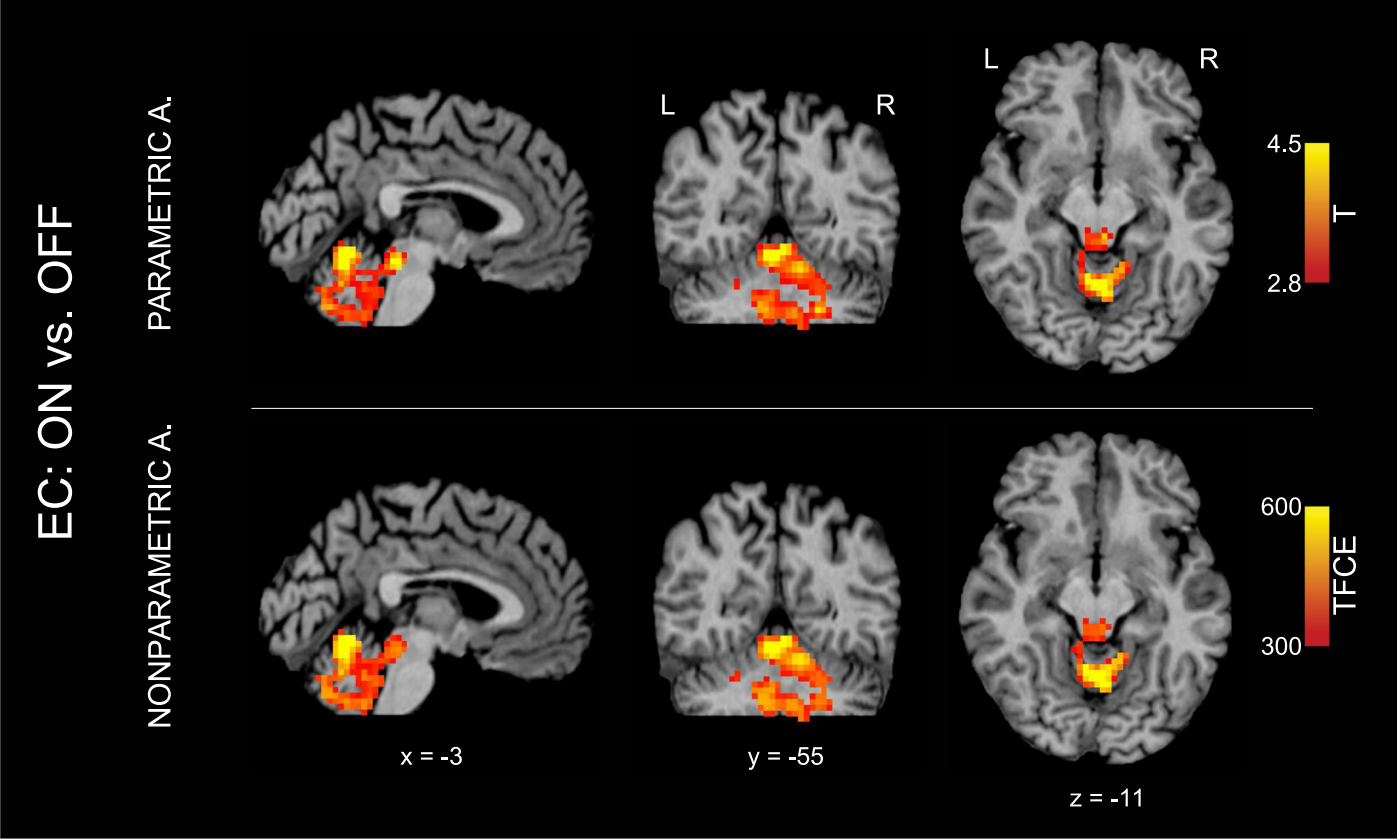
Resting-state fMRI connectivity increase of general connectivity in PD patients (*N* = 24) in the *ON* compared to the *OFF* condition. The general connectivity increase (eigenvector centrality analysis; contrast *ON* vs. *OFF* condition) in cerebellum and brainstem are shown with both parametric and nonparametric analysis (red-yellow clusters). Nonparametric analysis was performed using the threshold-free cluster enhancement (TFCE) technique with 10,000 permutations and a significance level of *p* < 0.05 (family-wise error corrected). Interestingly, the same result was obtained with both approaches. Note that all 16 local maxima obtained with the parametric analysis (see Table 2) were also detected as significant in the nonparametric analysis. A analysis, EC Eigenvector centrality

Table 2 presents a list of all local maxima of *T* scores within the cluster of general connectivity increase in cerebellar brain regions and brainstem after dopaminergic treatment. EC increase was obtained in the anterior and posterior lobe of the left and right cerebellum and particularly in lobuli V, VI, VIIIa, and IX. In the brainstem, local maxima were found in the pons and in the tegmentum. Figure 2 also shows this EC increase with orthogonal brain sections for several selected maxima (see first and fourth row, color-coded in red/yellow). The same EC increase was also obtained with the nonparametric approach and the TFCE toolbox with 10,000 permutations. Figure 3 shows a direct comparison between analyses (*p* < 0.05 with FWE-correction at the cluster level). All 16 local maxima obtained with the parametric analysis (see Table 2) were also found to be significant with the nonparametric analysis.

**Table 2:** Resting-state fMRI connectivity increase of general and selective connectivity in PD patients (*N* = 24) in the *ON* condition compared to the *OFF* condition*

Maximum		Structure	*x*	*y*	*z*	*T*	*p*	Corrected
1	Right cerebellum—AL, cerebellar nuclei		**9**	**− 46**	**− 26**	**5.74**	**0.00001**	***
		Right ventrolateral posterior thalamus	15	− 19	7	8.22	0.00001	**
		Left ventrolateral posterior thalamus	− 16	− 17	4	4.21	0.001	***
		Right mesencephalon (red ncl.)	5	− 23	− 9	3.91	0.001	***
		Left mesencephalon (red ncl.)	− 8	− 24	− 9	4.11	0.001	***
		Left globus pallidus	− 19	− 5	− 6	4.65	0.001	***
		Left SMA, BA6	− 21	− 22	55	6.29	0.000001	
		Left premotor, BA6	− 6	17	64	3.79	0.001	
2	Right cerebellum—AL, vermis (culmen), lobule V (92%)		**6**	**− 52**	**− 14**	**5.53**	**0.00001**	***
		Right subthalamus	9	− 13	− 5	3.27	0.01	
		Left subthalamus	− 6	− 10	− 5	4.22	0.001	
3	Right cerebellum—AL, vermis (culmen), lobule V (54%)		**3**	**− 55**	**− 11**	**5.48**	**0.00001**	***
		Right subthalamus	3	− 7	− 5	4.95	0.0001	***
		Left subthalamus	− 3	− 7	− 5	4.59	0.0001	***
		Right posterior and mediodorsal thalamus	11	− 27	6	4.32	0.0001	***
		Left posterior and mediodorsal thalamus	− 12	− 28	6	3.72	0.001	***
		Brainstem	11	− 26	− 26	3.85	0.001	***
4	Tegmentum		**− 3**	**− 31**	**− 14**	**5.45**	**0.00001**	***
		Right mesencephalon (substantia nigra)	8	− 19	− 17	3.86	0.001	***
		Left mesencephalon (substantia nigra)	− 7	− 20	− 17	3.64	0.001	***
		Right ventrolateral posterior thalamus	18	− 22	10	3.87	0.001	*
		Left ventrolateral posterior thalamus	− 13	− 22	12	4.23	0.001	***
		Left globus pallidus	− 18	5	− 8	4.24	0.001	
		Left SMA BA6	3	− 1	64	3.81	0.001	
		Left premotor	− 33	− 1	58	4.08	0.001	
5	Right cerebellum—AL, hemisphere (lingual), lobule V (86%)		**9**	**− 49**	**− 17**	**5.43**	**0.00001**	***
		Right ventral anterior thalamus	6	− 14	− 1	3.03	0.01	***
		Left ventral anterior thalamus	− 6	− 15	− 2	3.65	0.001	***
		Left globus pallidus	− 12	0	− 5	4.05	0.001	***
		Tegmentum	4	− 31	− 10	4.16	0.001	***
6	Left inferior posterolateral pons		**− 12**	**− 34**	**− 38**	**4.81**	**0.0001**	***
		Right posterior and ventrolateral posterior thalamus	15	− 20	9	4.44	0.0001	***
		Left posterior and ventrolateral posterior thalamus	− 15	− 21	9	4.16	0.001	***
		Left posterior putamen	− 29	− 10	− 1	4.23	0.001	***
		Left anterior putamen	− 20	6	−5	4.39	0.001	***
		Right globus pallidus	23	−5	−5	4.15	0.001	***
		Brainstem	6	−25	−28	3.63	0.001	***
7	Right cerebellum—PL, vermis, lobule IX (68%)		**6**	**− 58**	**− 41**	**4.59**	**0.0001**	***
		Right ventral thalamus	10	− 16	0	3.97	0.001	***
		Left ventral thalamus	− 9	− 16	− 2	3.84	0.001	***
		Pons	− 6	− 16	− 35	5.43	0.00001	***
8	Left cerebellum—PL, hemisphere (tonsil)		**− 21**	**− 55**	**− 41**	**4.35**	**0.0001**	***
		Left posterior thalamus (pulvinar)	− 15	− 28	10	4.00	0.001	
9	Left cerebellum—PL, hemisphere, nuclei, lobule VI (72%)		**− 12**	**− 55**	**− 20**	**4.28**	**0.0001**	***
		Left globus pallidus, putamen	− 12	5	8	4.19	0.001	
10	Left cerebellum—PL, vermis, lobule VIIIa (56%)		**− 6**	**− 67**	**− 38**	**4.27**	**0.0001**	***
		Right posterior and ventrolateral posterior thalamus	18	− 25	1	4.03	0.001	***
11	Right cerebellum—PL, vermis, lobule IX (56%)		**3**	**− 49**	**− 44**	**4.27**	**0.0001**	***
		Left SM1, BA 4	− 36	− 31	61	5.09	0.0001	
		Right SMA, BA 6	9	−25	61	3.96	0.001	
12	Left cerebellum—PL, vermis, lobule IX (78%)		**6**	**− 46**	**− 47**	**4.20**	**0.001**	***
		Right SM1, BA 4	18	− 28	64	3.66	0.001	
		Left SM1, BA 4	− 24	28	55	5.11	0.0001	
		Left globus pallidus, putamen	− 18	− 1	− 2	3.58	0.001	
13	Left cerebellum—AL, cerebellar nuclei		**− 9**	**− 46**	**− 32**	**4.12**	**0.001**	***
		Right anterior thalamus	6	− 4	1	3.43	0.001	
		Left anterior thalamus	− 3	− 4	1	3.00	0.01	
14	Left cerebellum—PL, hemisphere (tonsil), lobule IX (55%)		**− 9**	**− 58**	**− 44**	**4.07**	**0.001**	***
		Left ventrolateral posterior thalamus	− 15	− 22	7	4.73	0.0001	***
		Right ventral anterior thalamus	12	− 7	1	3.00	0.01	***
		Left ventral anterior thalamus	− 9	− 7	1	3.24	0.001	***
		Right posterior thalamus	16	− 27	− 2	3.00	0.01	***
		Left posterior thalamus	− 12	− 28	− 2	3.85	0.001	***
15	Left cerebellum—PL, vermis, lobule VI (50%)		**− 3**	**− 61**	**− 26**	**4.00**	**0.001**	***
		Right mediodorsal thalamus	12	− 18	6	3.88	0.001	
		Left ventrolateral thalamus	− 15	− 7	7	4.02	0.001	
16	Left cerebellum—AL, hemisphere (sup. quadrangular), lobule V (64%)		**− 12**	**− 49**	**− 11**	**3.97**	**0.001**	*
		Right posterior and ventrolateral posterior thalamus	18	− 19	7	4.48	0.0001	***
		Left posterior thalamus	− 12	− 31	7	3.93	0.001	***

*Maxima 1–16 (values in bold) denote the local maxima of general connectivity increase (based on the EC analysis) located in the cerebellum and brainstem after levodopa administration compared to condition without dopaminergic treatment. The analysis was restricted by mask involving expanded primary motor, premotor, sensorimotor cortex, basal ganglia, thalamus, brainstem, and cerebellum. Selective connectivity increase (based on correlation analysis) for all extra-cerebellar brain structures within the mask with each seed region in the *ON* as compared to the *OFF* condition is shown in non-bold values. All seed regions (maxima 1–16) are sorted according to the *T*-score in descending fashion. *x*, *y*, *z* local maxima of clusters in MNI coordinates derived from the contrast *ON* vs. *OFF* condition, *T T*-score, *p* uncorrected level of significance; *p < 0.05, **p < 0.01, ***p < 0.001—significance with family-wise error correction at cluster level; *AL* anterior lobe of the cerebellum, *PL* posterior lobe of the cerebellum

In addition to our EC findings with levodopa treatment, we observed the same EC increase when using a one-sample *t*-test across *ON*-*OFF* EC difference images (Fig.S1, top row). Remarkably, we obtained the same result regardless of adding the UPDRS-III tremor score as an additional covariate of no interest. The analysis was performed twice using the tremor score obtained with and without levodopa medication (see Fig. S1, middle and bottom row, respectively). Thus, the tremor score did not explain significant variance of EC change.

To investigate which brain regions are stronger connected to the brainstem and left and right cerebellar regions leading to a treatment-related increase in EC, additional seed-based connectivity analysis was performed. Here, we used all local maxima 1–16 obtained with the contrast *ON*-*OFF* to define the seed regions. To show pairwise differences with levodopa treatment, resulting correlation maps were fed into a statistical analysis with a paired *t*-test using the general linear model. In total, 16 group analyses were performed using paired *t*-tests in order to detect changes in selective connectivity with levodopa treatment using all 16 local maxima as seed regions. We obtained an increase of selective connectivity, that is, an increase of correlation of the BOLD time courses between cerebellum and subcortical regions as the thalamus, globus pallidus, and putamen and also in the brainstem (mesencephalon) (see local maxima 1–3, 5, 7–10, and 12–16 in Table 2 and also selective connectivity shown in Fig. 2). We also observed an increase of selective connectivity between cerebellum and cortical regions as the left and the right sensorimotor cortex and the supplementary motor area (see local maxima 11 and 12 in Table 2 and Fig. 2). Besides cerebellum, we found a treatment-related increase of selective connectivity between the tegmentum (see maximum 4 in Table 2 and Fig. 2) and many other motor regions as the left and the right mesencephalon (substantia nigra), the left and the right ventrolateral posterior thalamus, the left globus pallidus, the left supplementary motor area, and the left premotor cortex. We also detected increased connectivity between pons and subcortical structures such as thalamus, putamen, and globus pallidus (see maximum 6 in Table 2 and in Fig. 2). As described above, all 16 group analyses were repeated using a nonparametric approach using the TFCE toolbox. The permutation tests yielded the same results showing an increase of selective connectivity with levodopa treatment. This analysis confirms our findings obtained with the parametric approach.

Overall, we detected an increase of BOLD signal correlations between cerebellum and brainstem and other brain regions within the motor system in response to dopaminergic treatment. Note that we also investigated potential decreases of selective connectivity using all maxima listed in Table 2, however, without any significant result.

**Fig. 4:**
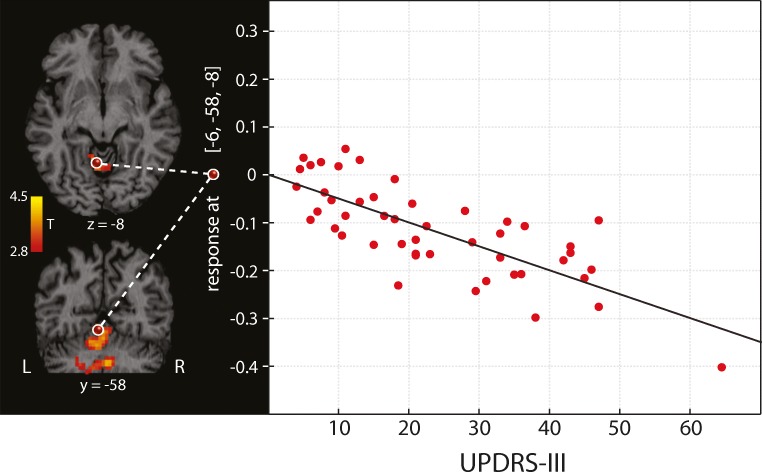
Negative correlation between the UPDRS-III motor score and the general connectivity (eigenvector centrality) in PD patients (*N* = 24) regardless of medication condition. The better the motor state of patients, the more connected is the cerebellum with the other motor regions in the network restricted by mask defined on Fig. 1. The dot plot demonstrates this relationship in a maximum selected from the correlation map (*p* < 0.05, family-wise error-corrected at the cluster level). Each patient is represented by two dots (with the *OFF* and the *ON* condition)

In addition to study intra-individual differences of general and selective connectivity using a paired design, we also performed a correlation analysis between EC and the total UPDRS-III score in order to investigate the relationship between connectivity and clinical symptoms. This correlation included all 48 EC maps of both the *OFF* and the *ON* session. We found a relationship between disease severity and general connectivity. In particular, a significant negative correlation between the UPDRS-III score and EC was obtained in the cerebellum (*p* < 0.05, FWE-corrected at the cluster level). The better the motor state of patients, the more are cerebellar regions connected with other brain areas of the motor system. Figure 4 shows these cerebellar regions (color-coded in red/yellow) and a dot plot for the global *T* score maximum.

### Motion Effects

The analysis of head motion during MR scanning yielded overall very subtle effects. The mean FD of all subjects showed a maximum of 0.7 mm. When excluding the subject showing the largest mean FD value, the mean FD was below 0.5 mm for all remaining subjects and sessions. When disregarding the 5% largest FD values, the maximum remaining FD was less than 2 mm, which is well below the nominal voxel dimension of our fMRI study. Only 17 out of 9552 frames from the entire study (i.e., 24 patients × 2 sessions × 199 image volumes) indicated single head movements by more than 2 mm, corresponding to < 0.2%. Moreover, there were no consistent differences in the motion parameters between the *OFF* and the *ON* condition. We note that we exclusively recruited patients of akinetic-rigid type in the current study but no tremor-dominant patients, which explains why motion-related bias was not an issue in this particular cohort.

## Discussion

We investigated brain connectivity alterations in PD patients related to acute treatment effect. Overall, the instant beneficial effect of levodopa on the clinical motor symptoms was related to the modulation of functional connectivity within the motor network. In sum, we found short-term general connectivity increases in the cerebellum and brainstem after the administration of a single levodopa dose. These were related to further selective connectivity increases between those regions and both cortical and subcortical motor structures. Interestingly, we found specific connectivity patterns between different cerebellar regions and the thalamus as well as different parts of the basal ganglia related to levodopa. Some of the connections are contralateral, some are ipsilateral, and some are bilateral. This multifaceted connectivity pattern is the major finding of our work showing the complexity of cerebellar interconnectedness in PD in response to levodopa treatment.

After years of neglect, the role of the cerebellum in PD has been recently brought to light [33]. The cerebellum is a complex and heterogeneous brain region, and recent studies in non-human primates have demonstrated that it forms an integrated bidirectional functional network with the basal ganglia [34, 35]. The modulatory activity of levodopa on resting-state basal ganglia networks has been tested in a double-blind placebo-controlled study in healthy subjects using seed-based analysis [36]. In line with our findings, levodopa mainly led to significant connectivity increases between the putamen and both the cerebellum and the brainstem in healthy subjects [36]. Additionally, connectivity reductions between ventral striatal and dorsal caudate seeds and default-mode network structures were also described in the same subjects. As for studies in PD patients, heterogeneous results have been reported concerning cerebellar connectivity during task-free fMRI. For example, Helmich and colleagues [37] did not report changes in connectivity between cerebellum and basal ganglia in de novo and mild PD patients (average disease duration 6.0 ± 0.6 years) as compared to controls. Wu and colleagues [38] reported increased regional homogeneity in the cerebellum of unmedicated early PD patients (disease duration 4.1 ± 1.8 years) as compared to controls. This increased connectivity was attenuated by levodopa intake. These authors also reported significant and extensive regional homogeneity reductions encompassing the basal ganglia, thalamus, and supplementary motor cortex. Applying the same connectivity analysis in early PD (disease duration 1.6 ± 1.1 years), a similar pattern was reported by Yang and colleagues [39], showing widespread changes in the patients group including regional homogeneity increases in the cerebellum and subthalamic nucleus and decreases in putamen and inferior frontal gyrus. Another study reported increases in cerebellar connectivity by means of resting-state fMRI in unmedicated PD patients in moderate disease stages (disease duration 6.6 ± 3.3 years) and subsequent normalization in the medicated state [40]. On the contrary, an opposite pattern, characterized by reduced cerebellar connectivity, was reported in advanced PD patient in the on state (disease duration 12.9 years) [41].

Considering these and our findings, we agree with Simioni et al. that the cerebellum might have a compensatory role in PD in mild-to-moderate stages, but in later stages, the progressive neurodegeneration would eventually hinder this mechanism [40]. Here, we report data in patients with moderate-to-advanced disease state where the endogenous compensatory mechanism might be already derailed. We thus hypothesize that the levodopa administration in PD re-establishes, at least in the short-term, the compensatory effect of the cerebellum. By means of correlation analysis, we showed that a higher interconnectedness of the cerebellum is paired with a better motor outcome. However, further studies, preferably with a longitudinal design, would be needed to verify this hypothesis. Increased resting-state connectivity between the basal ganglia and regions of the cerebello-thalamo-cortical network has been also reported by Helmich et al. as associated with tremor in PD [10]. However, our sample consisted of mainly akinetic-rigid patients, and differences in the pathophysiology of the two motor phenotypes are well known [42, 43]. Moreover, it has been proposed that the neural compensatory mechanisms to counteract akinesia and rigidity arising from striatal denervation might be the source of tremor in PD [10, 33, 42]. The compensatory role of the cerebellum in PD is also supported by a number of task-based fMRI studies, which have shown cerebellar hyperactivation in PD patients as compared to controls during simple movement execution and motor learning tasks [33].

Besides the cerebellum, we also found an interconnectedness increase following levodopa in the brainstem that was in turn selectively connected with subcortical (i.e., substantia nigra, ventrolateral posterior thalamus, and globus pallidus) and cortical (i.e., supplementary motor area) motor regions. Our findings emphasize the role of the brainstem in PD in context of brain pathology [44]. Interestingly, Hacker et al. previously identified impaired functional connectivity between the brainstem and the basal ganglia [41] with resting-state fMRI. In addition, diffusion tensor imaging studies reported selective alterations in the structural connectivity of the caudal portion of the substantia nigra that might also serve as an early PD biomarker [45]. According to our results, levodopa increases the functional connectivity within this crucial pathological network in PD, thus compensating, at least in the short-term, for the core pathological changes in PD. Note that our findings also show an increased cerebellar connectivity together with an improvement of motor performance as assessed by UPDRS-III score. In this respect, our results are in line with recent findings by Guan and colleagues [46] who reported a negative correlation between parameter estimates measured by ICA algorithm in the posterior cerebellum and motor impairments in the form of akinesia and rigidity. The comparison of our results with those of Guan et al. is justified since our cohort consisted of mainly akinetic-rigid PD patients and it is thus conceivable that the alterations in overall UPDRS-III highly reflect akinesia and rigidity. While mainly akinetic-rigid PD patients were included in our study, Dirkx and colleagues [47] investigated 15 patients of a tremor-dominant phenotype. In particular, they studied tremor-related connectivity patterns with and without levodopa medication showing that levodopa influenced tremor by acting on the cerebellar thalamus.

It has to be taken into consideration that we investigated the short-term effects following a single levodopa dose in a patient cohort in moderate-to-advanced PD stages. Thus, we cannot exclude that the prolonged use of dopaminergic therapy for several years might have induced long-term brain neuroplasticity phenomena that eventually influence the brain response to a single treatment dose [8]. A further limitation is the same *OFF*-*ON* order for all patients that might introduce a possible confound. We note that this has been a common limitation in similar previous studies [38, 48, 49], and we believe that our results within the motor network are predominantly related to the levodopa effect, consistent with our hypotheses. Moreover, in this study, we specifically investigated the effects of levodopa on the motor symptoms and on the brain motor network. However, it is now established that PD is additionally characterized by several non-motor features and by the impairment of other neurotransmitters besides dopamine [50]. Notably, the cerebellum is also functionally connected with prefrontal and posterior parietal associative brain regions, possibly mediating higher order cognitive functions [51, 52]. Further studies might thus focus on the potential impact from treatment-induced modulation of cerebellar functional connectivity on non-motor symptoms in PD. Additionally, future treatment strategies in PD should take into account that restoring the dopaminergic transmission within the nigrostriatal dopaminergic pathway might not be enough for treating the wide spectrum of different symptoms [14]. Finally, it has to be mentioned that the young age of our PD cohort might represent a limit for the generalizability of our findings to older patients. We would expect that individuals with later PD onset would present similar but less pronounced treatment-induced effects. However, this topic needs further investigation, because age does not only modulate the response to levodopa, but also the motor phenotype and the degree of dopaminergic dysfunction [21, 22, 53].

Taken together, our data indicate that the effect of levodopa on functional connectivity changes in particular in the cerebellum and brainstem may represent an important determinant in the modulation of clinical motor performance outcomes of PD patients as assessed by UPDRS-III and emphasize the notion of resting-state fMRI being a powerful tool for the investigation of treatment-related brain changes in PD.

## Electronic Supplementary Material

Figure S1 Resting state fMRI connectivity increase of general connectivity with levodopa treatment in PD patients (*N*=24) with and without tremor covariate (T.C.). The eigenvector centrality (EC) increase with levodopa treatment (*ON* vs. *OFF* condition) was obtained using a one-sample *t*-test over 24 EC difference images. In order to consider the tremor variability across patients, the analysis was performed using the tremor score of the UPDRS-III as an additional covariate. The Figure shows the EC increase without T.C. (top row), with using the T.C. without levodopa medication (*OFF* state T.C., middle row), and with using the T.C. with levodopa treatment (*ON* state T.C., bottom row). Note that we mainly recruited patients of akinetic-rigid type, and therefore, we obtained the same result in all three analyses. (PNG 813 kb)

High resolution image (EPS 6480 kb)
