# Supplementary figures and images for: Modulatory Effects of Levodopa on Cerebellar Connectivity in Parkinson’s Disease
Source: Cerebellum. 2018 Oct 8;18(2):212–24. doi: 10.1007/s12311-018-0981-y (PMC6443641; doi:10.1007/s12311-018-0981-y)

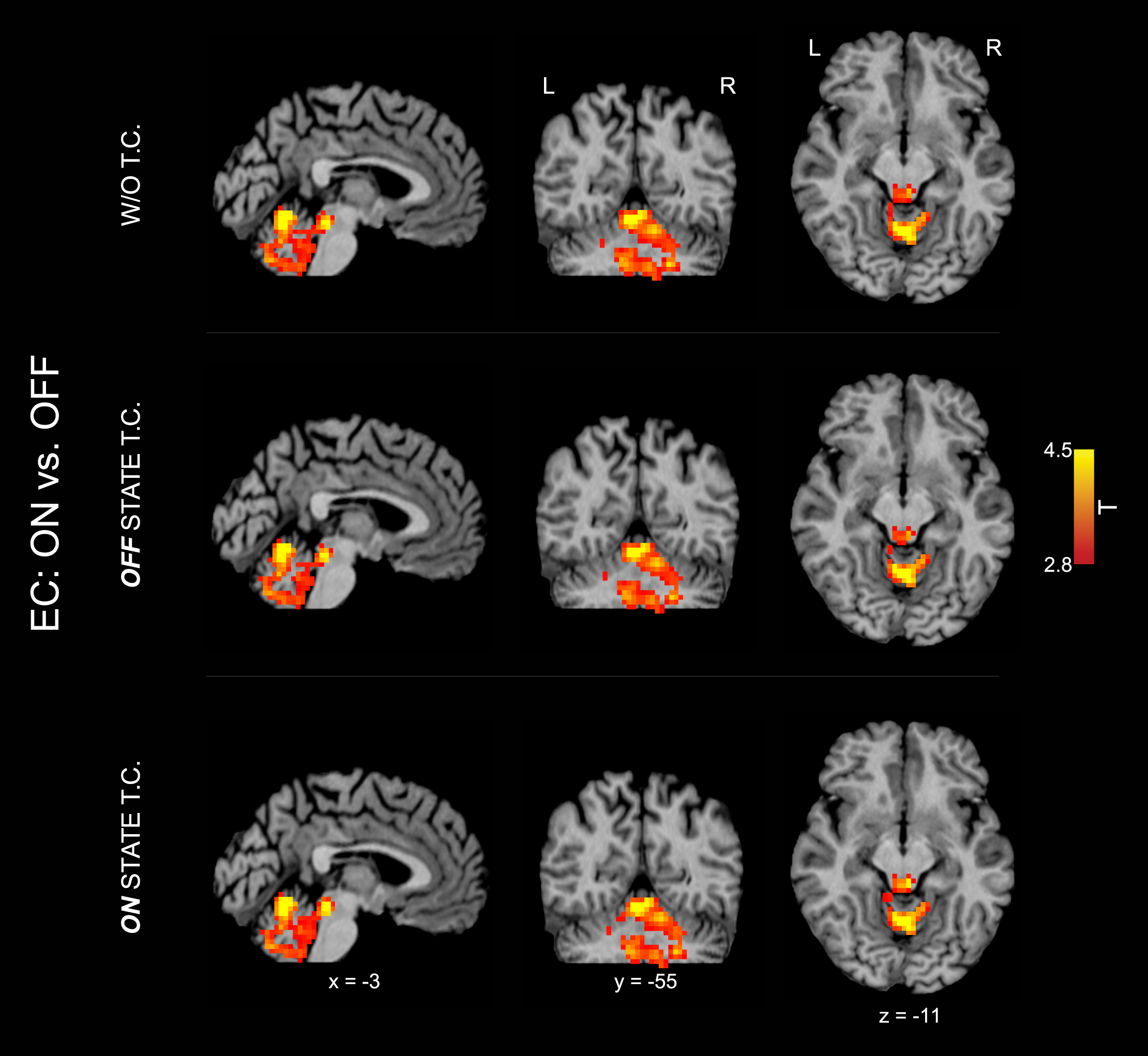

Supplement: Supplementary file 1 — Resting state fMRI connectivity increase of general connectivity with levodopa treatment in PD patients (N=24) with and without tremor covariate (T.C.). The eigenvector centrality (EC) increase with levodopa treatment (ON vs. OFF condition) was obtained using a one-sample t-test over 24 EC difference images. In order to consider the tremor variability across patients, the analysis was performed using the tremor score of the UPDRS-III as an additional covariate. The Figure shows the EC increase without T.C. (top row), with using the T.C. without levodopa medication (OFF state T.C., middle row), and with using the T.C. with levodopa treatment (ON state T.C., bottom row). Note that we mainly recruited patients of akinetic-rigid type, and therefore, we obtained the same result in all three analyses. (PNG 813 kb) [file 12311_2018_981_Fig5_ESM.png]
